# The microbiota of the surface, dermis and subcutaneous tissue of dog skin

**DOI:** 10.1186/s42523-020-00050-8

**Published:** 2020-09-22

**Authors:** Rocío García-Fonticoba, Lluís Ferrer, Olga Francino, Anna Cuscó

**Affiliations:** 1 Fundació Hospital Clínic Veterinari, Universitat Autònoma de Barcelona, Bellaterra, 08193 Barcelona, Spain; 2 Departament de Medicina i Cirurgia Animals, Universitat Autònoma de Barcelona, Bellaterra, 08193 Barcelona, Spain; 3 Servei Veterinari de Genètica Molecular, Departament de Ciencia Animal i dels Aliments, Universitat Autònoma de Barcelona, Bellaterra, 08193 Barcelona, Spain; 4 Vetgenomics, Ed. Eureka, PRUAB, Campus UAB, Bellaterra, 08193 Barcelona, Spain

**Keywords:** Skin microbiota, Epidermis, Dermis, Subcutaneous tissue, Next-generation sequencing, Dog

## Abstract

**Background:**

Studies using highly sensitive molecular techniques have detected bacterial communities below the human epidermis. Depending on their abundance and composition, this finding could be clinically relevant. The aim of this study was to determine if bacteria can be detected in the dermis and subcutaneous tissue of dogs without cutaneous disease using two different approaches: traditional cultures and DNA sequencing of the V4 region of bacterial 16S rRNA gene using next-generation sequencing (NGS).

**Results:**

Seven healthy dogs were included in the study, and two sets of samples were collected from each subject. Sample sets were composed of a 6-mm abdominal skin biopsy, including epidermis, dermis, and subcutis, a skin surface swab, and an environmental blank sample for contamination control. One set of samples from each dog was submitted for bacterial culture and the other one for bacterial DNA amplification and sequencing. Five different bacterial genera (*Staphylococcus*, *Bacillus*, *Corynebacterium*, *Streptococcus,* and *Enterococcus*) were isolated in five out of the seven skin surface swab samples with aerobic microbiological culture methods, while no growth was obtained from the other two samples. Although some DNA could be amplified from epidermal, dermal, and subcutaneous tissue samples, the results of the NGS were similar to those of the blanks.

**Conclusion:**

When investigated with aerobic microbiological culture methods, the dermis and subcutaneous tissue of dogs are sterile. NGS techniques lead to the detection of some bacterial DNA, similar to the signal detected in blanks, which does not support the presence of a microbiota in dermis or subcutaneous tissue.

## Background

The study of the cutaneous microbiota has aroused enormous interest in the last decade [1–3]. Numerous studies have revealed the complexity of the microbial skin communities of humans, dogs, and cats [4–6]. Multiple interactions between the skin microbiota and the immune system have also been demonstrated, so that a diverse and balanced microbiota is considered an essential component of a healthy skin [4, 7, 8]. Furthermore, identifying and characterizing the cutaneous microbiota can help us to understand how microbial diversity may contribute to cutaneous diseases, and to elucidate the roles of different microorganisms in the pathogenesis of dermatological conditions such as atopic dermatitis [8–10].

Culture-based techniques have been useful to detect bacterial populations in the epidermal surface but have failed to detect microorganisms in the dermis and subcutaneous tissue [11, 12]. This explains why skin microbiota has been traditionally assumed to be restricted to the epidermal surface and appendageal structures, while dermis and subcutaneous tissue have been considered sterile. However, some organisms may not be easily detectable with conventional culture methods due to many factors [13]. The use of next-generation sequencing (NGS) techniques to characterize microbial communities has increased in recent years, opening the possibility of readily identifying bacteria that may be elusive to culture-based studies [14, 15].

Using NGS and other molecular approaches, the group of Nakatsuji changed the paradigm of a sterile dermis and subcutaneous tissues [16]. These authors were able to identify a small bacterial community in the dermis and superficial subcutaneous adipose tissue of healthy humans. A subsequent study confirmed the existence of a bacterial community in the dermis and revealed significant differences in microbial composition when comparing epidermal and dermal compartments [11].

The existence of dermal microbiota is of doubtless physiological importance, because these microorganisms can play a role in the regulation of the delicate immune responses of the skin. Moreover, depending on their abundance and composition, the presence of a dermal or subcutaneous microbiota could also be clinically relevant [11, 16]. In animals with cutaneous lesions, skin biopsies are commonly investigated with cultures and molecular techniques to detect and identify microorganisms that could have an etiologic role [17–20]. An initial assumption in these investigations is that the dermis and subcutaneous tissues of healthy animals are sterile.

Although some authors have investigated the dermal and subcutaneous microbiota of dogs with pyogranulomatous dermatitis and panniculitis [21] and in dogs with mast cell tumors [22], the composition of the microbiota of the different layers of the skin (epidermis, dermis, and subcutaneous tissue) of dogs without cutaneous disease has not been studied.

The primary aim of this study was to determine if bacteria can be detected below the canine epidermis (dermis and subcutaneous tissue) using two different approaches: aerobic microbiological cultures and microbiota profiling using DNA sequencing of the V4 region of the bacterial 16S rRNA gene. Studies in low-biomass samples have to be performed very carefully since the samples are prone to contamination with exogenous DNA, leading to false results [23–25]. To address this risk, we included three different blank samples to detect any potential environmental or kit contamination. The secondary aim of this study was to compare the microbial profiles obtained using aerobic microbiological culture methods with those obtained with NGS in skin samples of healthy dogs.

## Results

**Fig. 1:**
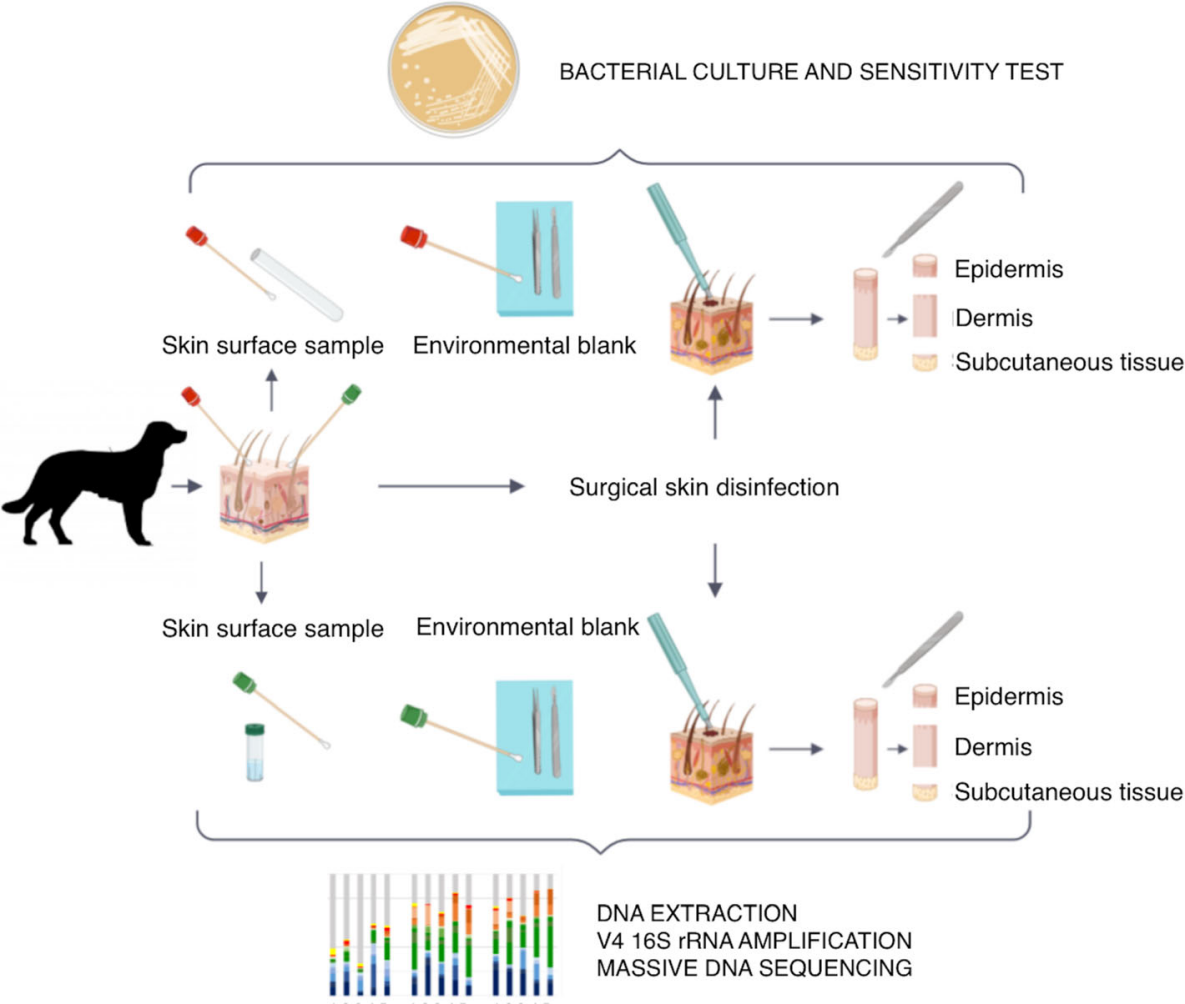
Collection of samples. Seven dogs were included in the study. Two sets of samples were collected from each subject. Samples sets were composed of a skin surface swab taken prior to surgical skin disinfection, a 6-mm abdominal skin biopsy, including epidermis, dermis, and subcutis, and an environmental (operating room) blank sample. One set of samples from each dog was submitted for bacterial culture and the other one for bacterial DNA amplification and sequencing. See “Animals and study design” of “Methods” section for more detailed information

A total of seven healthy, privately-owned dogs (4 intact females and 3 neutered males) without previous history of skin disease were included in the study. Their ages ranged from 1.5 to 14 years (mean age: 6.3 years) (Additional file 1). Two sets of samples were collected from each subject. Sample sets were composed of a skin surface swab taken prior to surgical skin disinfection, a 6-mm abdominal skin biopsy, including epidermis, dermis and subcutis, and an environmental (operating room) blank sample. One set of samples from each dog was submitted for bacterial culture and the other for bacterial DNA amplification and sequencing (Fig. 1).

### Bacterial cultures

Bacterial cultures from swabs of abdominal skin surface yielded 11 isolates from 5 of the 7 dogs, while no bacterial growth was obtained from skin surface samples of the other two dogs. Bacterial isolates included *Streptococcus* sp*.*, *Staphylococcus* sp*.*, *Corynebacterium* sp*., Bacillus cereus,* and *Enterococcus* sp. (Table 1).

**Table 1:** Number of isolated strains of bacteria from skin surface samples

BACTERIAL ISOLATES	NUMBER OF COLONIES						
	Dog1	Dog 2	Dog 3	Dog 4	Dog 5	Dog 6	Dog 7
Streptococcus sp	0	4	0	0	0	0	2
Staphylococcus sp	0	1	13	0	2	0	0
Corynebacterium sp.	0	0	0	25	3	0	0
*Bacillus cereus*	0	0	4	1	0	0	4
Enterococcus sp	0	0	7	0	0	0	0

Cultures of samples from epidermis, dermis, and subcutaneous tissue were negative in all cases. These samples were taken from abdominal skin, following surgical skin disinfection and under sterile conditions. An environmental blank sample was also cultured during each biopsy procedure, in all cases negative.

### Microbiota analysis

All the samples, including the blanks, presented DNA amplification and were sequenced, even when the bacterial cultures were negative. We evaluated microbiota diversity and composition in the skin surface swabs, the environmental blank, and the biopsy samples, obtained after surgical skin disinfection, corresponding to epidermis, dermis, and subcutaneous tissue. After V4 16S rRNA amplification, skin surface samples presented the largest quantity of DNA when compared to inner skin layers and blank samples (Additional file 2).

#### Microbiota diversity

**Fig. 2:**
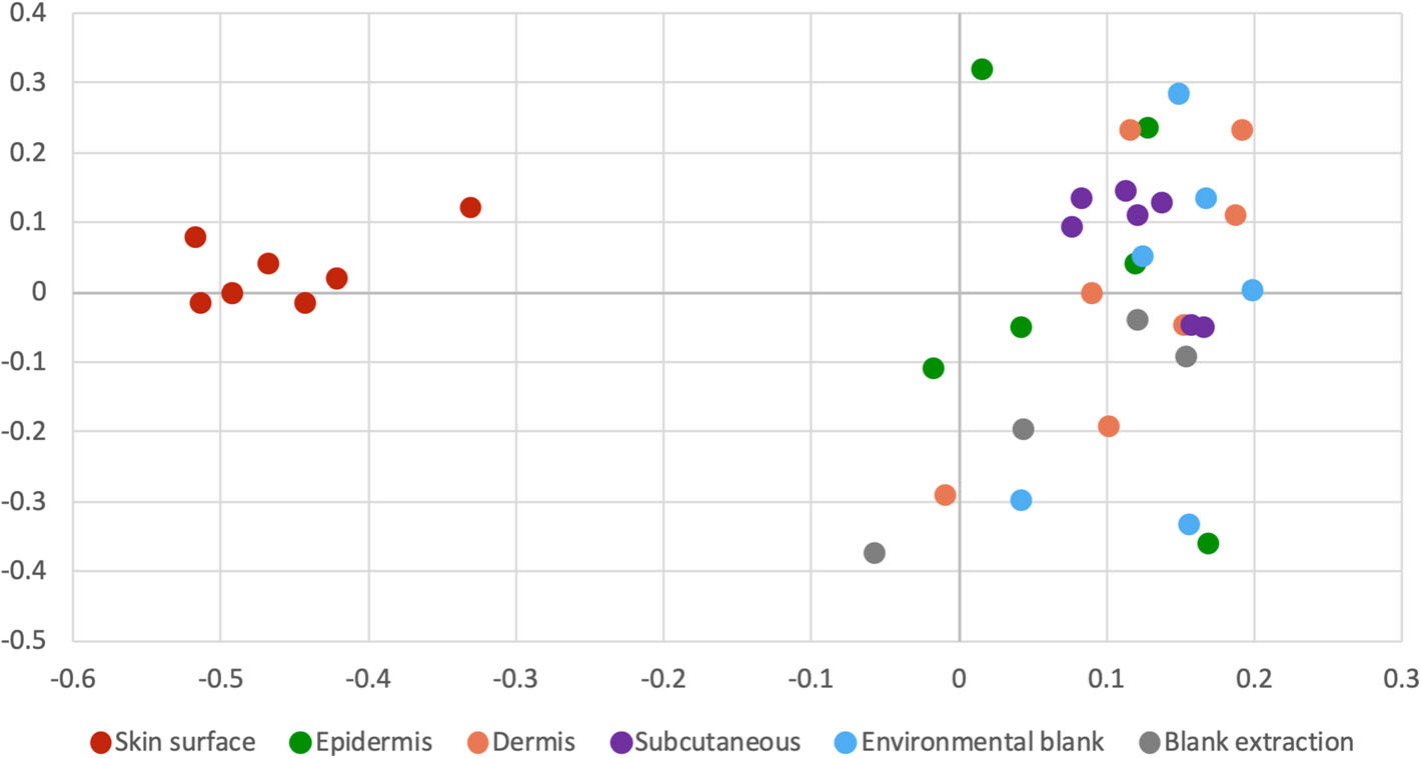
Principal coordinate analysis plots of Bray-Curtis distance matrices. Clustering of skin surface samples displaying similarity in abundance and composition of bacterial 16S rRNA gene sequences contained in these samples. Clustering of cutaneous skin layers and blanks reveals similarities in their microbial communities

To evaluate the overall microbial community structure and similarity between samples (beta diversity) in terms of abundance and composition, we used a Bray-Curtis distance matrix. The associated Principal Coordinates Analysis (*PCoA*) plot shows that skin surface samples clustered separately from the rest of the samples (Fig. 2). We observed similar trends, when analyzing Unweighted and Weighted UniFrac PCoA plots (Additional file 3). Confirming this observation, PERMANOVA pairwise comparisons were significant between skin surface and all the skin layers and blanks (*p*-value < 0.05). Moreover, extraction blanks –but not environmental blanks– were statistically different from epidermis (Unweighted UniFrac), dermis (Weighted UniFrac), and subcutaneous tissue (Bray Curtis and Weighted UniFrac) (Additional file 4). This implies that, in qualitative and quantitative terms, the skin surface samples, obtained before surgical scrubbing, have a microbiota composition very different from the epidermis, dermis, and subcutaneous tissue, the environmental blank, and the extraction blank.

**Fig. 3:**
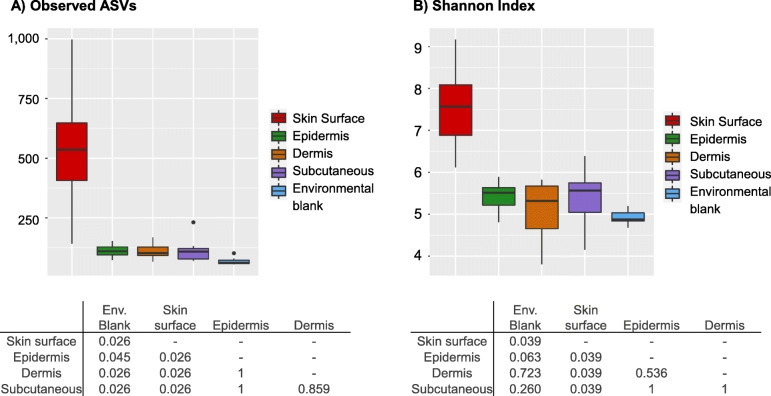
Alpha diversity analysis of the skin surface, skin layers, and environmental blanks. Observed ASV (A) and Shannon index (B) were evaluated. Boxplots represent alpha diversity values at 6000 sequences/sample. Statistical comparisons were performed at this depth using a paired Wilcoxon test. *P*-value < 0.05 is considered significant

To evaluate microbiota diversity within samples, we used two alpha diversity metrics: observed ASVs (richness) and Shannon diversity index (richness and evenness) (Fig. 3). For both indexes, samples from skin surface presented the greatest diversity and were significantly different from the skin layers (epidermis, dermis, subcutaneous tissue) and blanks. Epidermis, dermis, and subcutaneous tissue shared similar values and were not significantly different from each other. The skin layers presented significantly higher numbers of observed ASVs than blanks, but the difference could not be confirmed using the Shannon Index.

#### Microbiota composition

**Fig. 4:**
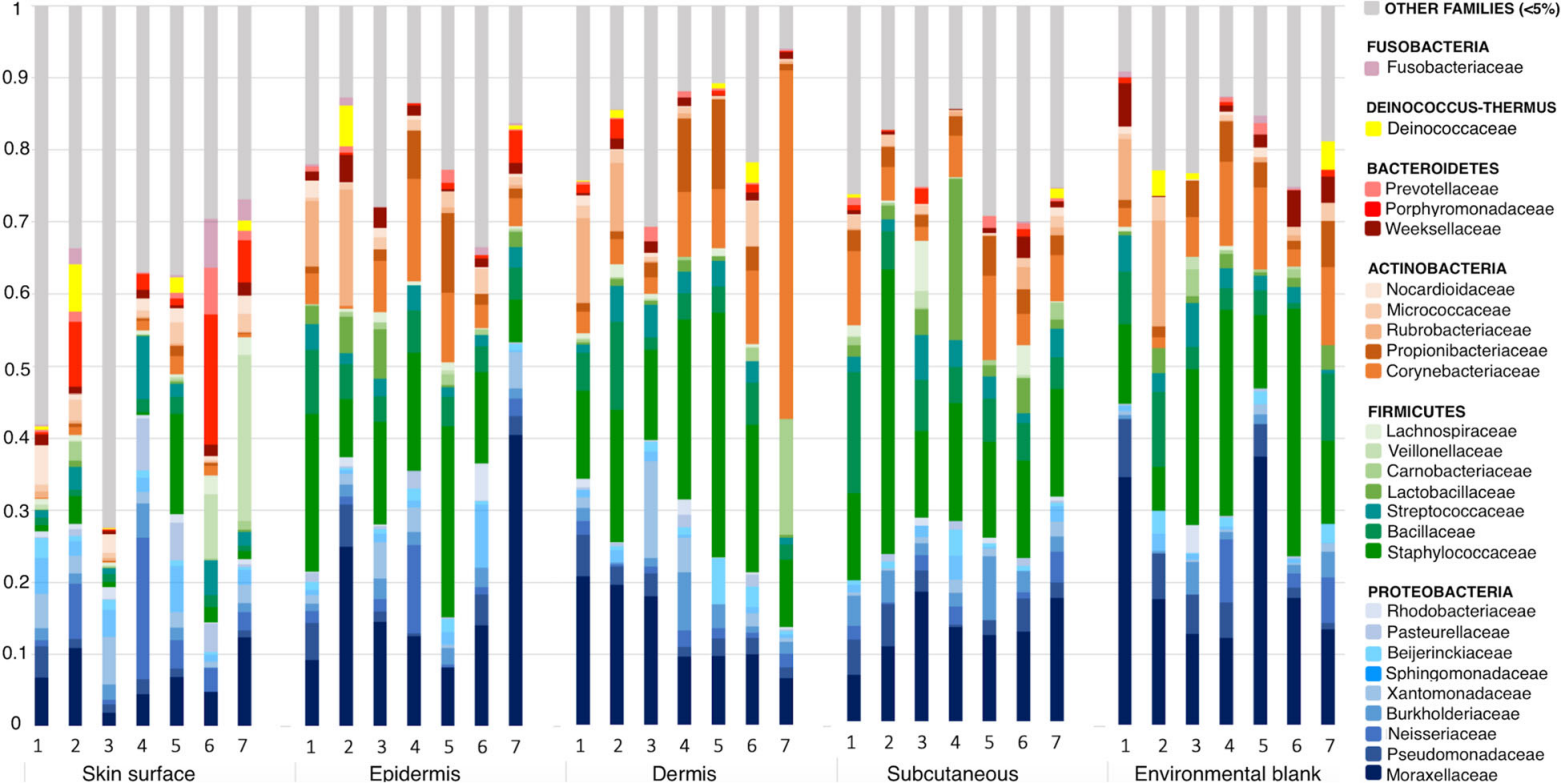
Relative abundance of most abundant bacterial taxa per dog and type of sample. High variability is observed between dogs, with samples from skin being the most variable. Despite individual variability, samples from all skin compartments are similar to each other, and also in comparison to environmental blank samples. Families with less than 5% relative abundance are represented in grey (Other families)

The samples from skin surface swabs showed a high degree of variability between dogs in terms of abundance of bacterial taxa. In contrast, samples from all other skin compartments and blanks were very similar to each other (Fig. 4). Some of the microbial families were found ubiquitously in both skin samples and environmental blanks.

The most abundant phyla identified in samples from skin surface were Proteobacteria, followed by Actinobacteria, Bacteroidetes, and Firmicutes. Moraxellaceae was the most common family, followed by Nocardioidaceae, Xanthomonadaceae, and Sphingomonadaceae.

On samples from epidermis, dermis, and subcutaneous tissue, the most abundant phyla were Firmicutes, Proteobacteria, Actinobacteria, and Bacteroidetes. Similar findings were obtained when sequencing environmental blanks, where Firmicutes and Proteobacteria were the predominant phyla.

Additional file 5 shows the final ASV table for all the samples, after removal of contaminants (see Methods).

#### Microbiota negative controls

When evaluating the negative controls, some trends can be elucidated. We identified two clear PCR contaminants: the families *Halomonadaceae* and *Shewanellaceae*. The higher the input DNA mass, the lower the concentration of the contaminants. As previously seen, skin swabs were the samples that presented more DNA amplification and positive microbiological cultures, and present lower *Halomonadaceae* and *Shewanellaceae* values (Additional file 6). Decontam R package allowed us to detect extra contaminants representing lower relative abundance such as several genera of the Burkholderiaceae family (*Variovax*, *Ralstonia*, *Achromobacter*, *Aquabacterium*), among others (Additional file 7).

## Discussion

In this study, traditional culture-based methods and V4 16S rRNA NGS were used to investigate the presence of a microbiota in the dermis and subcutaneous tissue of dogs with no cutaneous disease.

Bacterial growth was obtained from samples of the skin surface in five of the seven dogs, while no growth was observed from epidermis, dermis, subcutaneous tissue, or environmental blanks. These results are clinically relevant, as they indicate that, when investigated with aerobic microbiological culture methods, the dermis and subcutaneous tissue of healthy dogs are considered sterile.

Eleven isolates from five bacterial genera grew from the skin surface samples, including *Streptococcus* sp*.*, *Staphylococcus* sp*.*, *Corynebacterium* sp*., Enterococcus* sp., and *Bacillus cereus.* This contrasts with the number of families detected in these samples using V4 16S rRNA gene sequencing. Furthermore, the isolated bacteria were not necessarily the ones with a greater abundance in the NGS analysis. Thus, culture-based methods overrepresent the relative abundance of some easily culturable species while underestimating the real microbiota diversity detected using NGS. A delicate balance among temperature, nutrients, incubation time, and atmosphere determines the success of bacterial cultures [26]. The standard culture methods present the ideal conditions just for a minority of the bacteria that compose the cutaneous microbiota [13]. In this study, we performed only aerobic cultures incubated for 48 h at 37 °C. Nor were enriched media employed; under such specific conditions, selection and growth pressure exist for certain bacteria to the detriment of others.

In general, the cultured bacteria from the skin swabs were also identified in the NGS microbiota analysis, which agrees with previous studies [14, 27]. One exception was the *Enterococcus* sp. that was isolated from skin surface samples of dog 3 but was not detected in the microbiota analysis. Failure of NGS to identify culturable bacteria, although previously reported [14], is challenging to justify. The growth obtained using traditional culture methods may be a false positive due to contamination [28] or the consequence of low specificity of the V4 primer [29].

The NGS approach made it possible to characterize the skin surface microbiota and compare it with the amplification and sequencing of the epidermis, dermis, and subcutaneous samples, as well as the environmental blank, for each dog. The skin surface had a rich and diverse microbiota. It was found that Proteobacteria, Actinobacteria, Bacteroidetes, and Firmicutes were the most common phyla observed in the skin surface samples, concordant with previous studies in healthy dogs [5, 9]. In contrast, samples from the epidermis, dermis, and subcutaneous layers showed less richness and diversity and were more similar to the blanks. These results suggest that most of the taxa identified in epidermis, dermis, and subcutaneous tissue are probably contaminants. However, further investigation, including more animals, will be needed to determine whether any low-abundance taxa could be inhabiting the inner layers, as suggested by the significant differences in observed ASVs and by the non-significant differences in Shannon index when comparing skin layers and environmental blanks.

It is essential to underline that the inner layers of the skin are low-biomass samples, and prone to exogenous bacterial DNA contamination, which can significantly influence ﻿the resulting microbial community profile [23, 24]. Sample contamination can have several sources, either during sample collection or during sample processing, microbial DNA extraction, PCR amplification, and sequencing [24]. In the present study, three negative controls were processed with the samples to assess contamination during sample collection (environmental blank), DNA extraction (extraction blank), and PCR amplification (NTC).

There are some limitations to this study. First of all, the number of dogs included in the study. Considering that the skin microbiota is highly variable, we may have missed significant differences by looking at this number of animals. Larger cohorts are needed to evaluate if any low-abundant bacteria may inhabit in the skin layers. Secondly, the separation of the skin into its three compartments (epidermis, dermis, and subcutaneous tissue) was done by naked-eye dissection, without the help of magnification instruments, as in the study of Nakatsuji *el a*l. It was done in this way because this is the most commonly used method in clinical dermatology when investigating the dermis and subcutaneous tissue microbiologically [30]. Additionally, it is necessary to highlight that in the present study the existence of a microbiota below the epidermis has been investigated using only aerobic cultures and NGS. Perhaps using other highly sensitive techniques, such as in situ fluorescence used by Nakatsuji et al. [16] would have increased the probability of detecting bacterial populations that were found in very small numbers. Finally, only abdominal skin samples were included to ensure homogeneity of the samples in the comparison. Different body regions have variations in anatomy and physiology, including different number of hair follicles and various types and abundance of glands. It is possible that these differences could modify the ability to sustain microbial life. Further research would be needed to asses this point.

## Conclusions

In the present study, no bacteria grew from the epidermis, dermis, and subcutaneous tissue of dogs when using aerobic microbiological culture methods. Conversely, some DNA in an amount similar to the signal detected in blanks, could be amplified and sequenced from the inner skin layers in the NGS approach. All together, these results reveal the absence of a clearly defined microbiota in the inner layers of dog skin. Further analyses are needed to determine whether some specific low-abundant bacteria may inhabit these regions.

## Methods

### Animals and study design

A total of seven privately-owned dogs without previous history of infectious or inflammatory skin disease were included in the study (Additional file 1). Their age ranged from 1.5 to 14 years (mean age: 6.3 years). There were 4 intact female dogs and 3 neutered male dogs. The animals were not treated with antibiotics, anti-inflammatory agents, or immunosuppressive drugs for at least 4 weeks prior to sample collection or with topical treatments or antiseptic bathing during at least 2 weeks prior to sample collection. The dogs were to undergo different surgical procedures (ovariectomy, splenectomy and resection of abdominal cystic mass, and removal of benign skin tumours) and samples were taken immediately before the interventions. The owners gave written consent to the collection of samples, and the hospital’s research committee approved the procedure.

Two sets of samples of healthy abdominal skin were collected from each dog, one for microbiological culture and the other for microbiota sequencing. Each set was composed of swab samples (from environment and skin surface) and one cutaneous biopsy that was divided into epidermis, dermis, and subcutaneous tissue (Fig. 1).

For the traditional bacteriological culture, the skin surface was sampled using a cotton sterile swab (Deltalab; *Rubí, Spain*), which was deposited on a modified Stuart transport-medium until culture. For microbiota sequencing, we sampled using a sterile nylon swab (Zymo Research, Irvine, CA), which was introduced in DNA/RNA Shield lysis tubes (Zymo Research, Irvine, CA) and kept at 4 °C until sequencing. In both cases, prior to sampling swabs were moistened with sterile 0.9% sodium chloride solution (FisioVet®*, B.Braun Melsungen AG; Mesulgen, Germany*) and then rubbed for 60 s over a 4 cm^2^ area of skin. Also, sterile swabs were deposited on the sterilized surgical field while the samples were obtained and processed (environmental blank samples).

After collecting the skin surface samples, the skin biopsies were obtained. For surgical skin preparation, hair was removed using a disinfected electric clipper, and, subsequently, skin was rubbed using gauze impregnated in a soap solution *based on* 4*%* chlorhexidine digluconate *(ClorhexVet®, B.Braun Melsungen AG; Mesulgen, Germany).* After that, the skin was scrubbed according to a standardized protocol based on 1% chlorhexidine digluconate aseptic solution scrubs *(Desinclor® 1%, Imark; Ajalvir, Madrid, Spain)* alternated with 96% alcohol. This procedure was repeated 3 times, with a 30-s contact time in each step.

After surgical skin disinfection, two 6-mm biopsies were obtained under sterile conditions from an area of the abdominal skin close to the site where skin surface swabs were taken. Each skin biopsy was sectioned into epidermis, dermis, and subcutis on a sterile surgical field to avoid microbial contamination during the process. Each sample was dissected using sterile surgical material. To minimize sample cross-contamination, gloves and sets of surgical material were replaced between the processing of each sample.

Specifically, epidermis was obtained by sectioning a layer approximately one millimeter thick from the outermost part of the biopsy sample with a scalpel blade. Then, the outermost 3 mm of the sample were dissected and classified as dermis, while the rest of the sample was considered subcutaneous tissue.

To evaluate the skin microbiota in deeper layers, we considered three types of negative controls: i) at sample collection, a swab was placed on the working surface during the whole process to assess the environmental contamination; ii) at DNA extraction, a swab without any sample was processed with the samples to assess the potential contamination of the DNA extraction kit; iii) at the PCR step, a PCR without any sample was processed to assess the potential contamination of the PCR kit.

### Bacterial culture

Samples for aerobic bacterial culture (environmental blank, skin surface swab, and epidermal, dermal, and subcutaneous tissue) were sent in transport medium to Leti® Animal Health Laboratories (Barcelona, Spain) ﻿and processed within 2 days after collection. Streak plating from each sample on blood agar, MacConkey agar, and Sabouraud Chloramphenicol agar was performed, and then the sample was incubated at 37 °C for 48 h.

### DNA extraction

Skin swab and biopsy samples were collected and kept at 4 °C in RNA/DNA Shield lysis tubes (Zymo Research, Irvine CA) until the DNA extraction. Bacterial DNA was extracted using the ZymoBIOMICS™ DNA Microprep Kit (Zymo Research, Irvine CA) following the manufacturer’s instructions. DNA samples (20 μl) were stored at − 20 °C until further processing. To assess for contamination from the laboratory or reagents, a blank sample with a sterile swab tip was processed for each extraction batch. Total DNA was quantified using Nanodrop.

### V4 16S PCR amplification and sequencing

The hypervariable V4 region of 16S rRNA gene was amplified for each sample and negative control. A negative template control (NTC) for the PCR was also included. The forward primer was composed of the adapter linker, the key, the barcode (different for each sample), and the forward primer 515F (5′-GTGYCAGCMGCCGCGGTAA-3′). The reverse primer was composed of the adapter linker and the R806 reverse primer (5′-GGACTACNVGGGTWTCTAAT-3′). The Phusion Hot Start II High-Fidelity DNA polymerase (Thermo Fisher Scientific, Waltham, MA) was used to amplify the V4 16S rRNA gene. PCR mixture (25 μl) contained 2.5 μl of DNA template, 5 μl of 5× Phusion® High Fidelity Buffer, 2.5 μl of dNTPs (2 mM), 0.5 μM of each primer, and 0.5 U of Phusion® Hot Start II Taq Polymerase. The thermal profile consisted of an initial denaturation of 30 s at 98 °C, followed by 30 cycles of 15 s at °98 C, 15 s at 55 °C, 20 s at 72 °C, and a final extension of 7 min at 72 °C. After DNA purification using an Agencourt AMPure XP kit (Beckman Coulter, Brea, CA) with a ratio 1:1, quality and quantity of PCR products were determined using Agilent Bioanalyser 2100 (Agilent, Santa Clara, CA) and Qubit fluorometer (Thermo Fisher Scientific, Waltham, MA).

The pool containing barcoded samples and negative controls was sequenced on an Ion Torrent Personal Genome Machine (PGM) with the Ion 318 Chip Kit v2 (Thermo Fisher Scientific, Waltham, MA) following the manufacturer’s instructions.

### Quality control of the sequences and ASV filtering

Raw reads were demultiplexed and imported into the Quantitative Insight Into Microbial Ecology 2 (QIIME 2) software program [31], which was used to analyze the whole dataset. DADA2 was used as quality filtering method in order to denoise, dereplicate single-end sequences, and remove chimeras [32]. The forward primer was removed, and the sequences were truncated at a length of 220 bases. Amplicon Sequence Variants (ASVs) were used to classify the sequences and assign taxonomy using SILVA release 132 [33] at 99% of similarity and trimmed to the V4 region with the primers used, as reference database.

With the initial ASV table, we removed chloroplasts and mitochondria sequences since they are not bacterial members of the microbiota. Moreover, Shewanellaceae and Halomonadaceae families were also removed, since they presented the highest abundance in the NTC sample (Additional file 6).

After this first filtering step, we applied decontam R package [34] to remove other contaminants. Since these are low-biomass samples, we applied the prevalence method as recommended by the authors. Since different environments need to be decontaminated differently (especially when there are differences on initial biomass) [34], we applied the software three times: i) to skin swab samples and blanks, ii) to the biopsies and blanks, and iii) to the whole dataset. Contaminants that appeared TRUE in the biopsies approach or in two out of the three approaches were removed from the final ASV table (Additional file 7).

### Taxonomy and diversity analysis

For carry out diversity analyses, we worked at the sequencing depth of 6000 sequences/sample. With the final filtered ASV table (Additional file 5), alpha diversity was calculated using observed ASVs and the Shannon Index. Beta diversity was assessed with Bray-Curtis, Weighted UniFrac and Unweighted UniFrac.

Statistical significance of the alpha diversity values between skin samples groups (skin surface, epidermis, dermis, subcutaneous tissue, and environmental blank) was assessed using a pairwise Wilcoxon test (paired) in R, and *p*-values were corrected for FDR. Statistical significance of the beta diversity clustering by skin region was assessed using PERMANOVA.

## Supplementary information

**Additional file 1.** Metadata for dogs included in the study.

**Additional file 2.** Concentration after 16S rRNA V4 amplification and sequencing depth of each sample.

**Additional file 3.** Unweighted and Weighted UniFrac PCoA plots.

**Additional file 4.** PERMANOVA pairwise comparisons on beta diversity metrics.

**Additional file 5.** Final ASV table (contaminants removed).

**Additional file 6 **Boxplot representing the relative abundance of *Halomonadaceae* and *Shewanellaceae* on the different sample types.

**Additional file 7.** Contaminants detected using Decontam R package. Criteria for removing are detailed in Material and methods.

## Data Availability

The raw sequences have been submitted to NCBI under Bioproject PRJNA642025.

## Abbreviations

PCoA: Principal coordinates analysis
rRNA: Ribosomal RNA
NGS: Next-generation sequencing
ASV: Amplicon sequence variant
