# Supplementary material for: The microbiota of the surface, dermis and subcutaneous tissue of dog skin
Source: Anim Microbiome. 2020 Sep 22;2:34. doi: 10.1186/s42523-020-00050-8 (PMC7807805; doi:10.1186/s42523-020-00050-8)
Supplement: Supplementary file 1 — Additional file 1. Metadata for dogs included in the study. [file 42523_2020_50_MOESM1_ESM.pdf]

Additional file 1: Metadata for dogs included in the study.

Case no.	Age<br>years	Weight<br>Kg	Sex	Breed	Surgical procedure
1	2	9	Female	Mixed breed	Ovariectomy
2	15	12	Neutered male	Jack Russell Terrier	Cryotherapy for removal of multiple sebaceous adenomas
3	1.5	16	Female	Border collie	Ovariectomy
4	1.5	21	Neutered male	Mixed breed	Resection of abdominal cystic mass
5	2	26	Female	Golden retriever	Ovariectomy
6	13	4	Neutered male	Yorkshire terrier	Splenectomy
7	9	15	Female	Beagle	Lipoma resection
