# Supplementary figures and images for: The microbiota of the surface, dermis and subcutaneous tissue of dog skin
Source: Anim Microbiome. 2020 Sep 22;2:34. doi: 10.1186/s42523-020-00050-8 (PMC7807805; doi:10.1186/s42523-020-00050-8)

# PCoA Weighted UniFrac

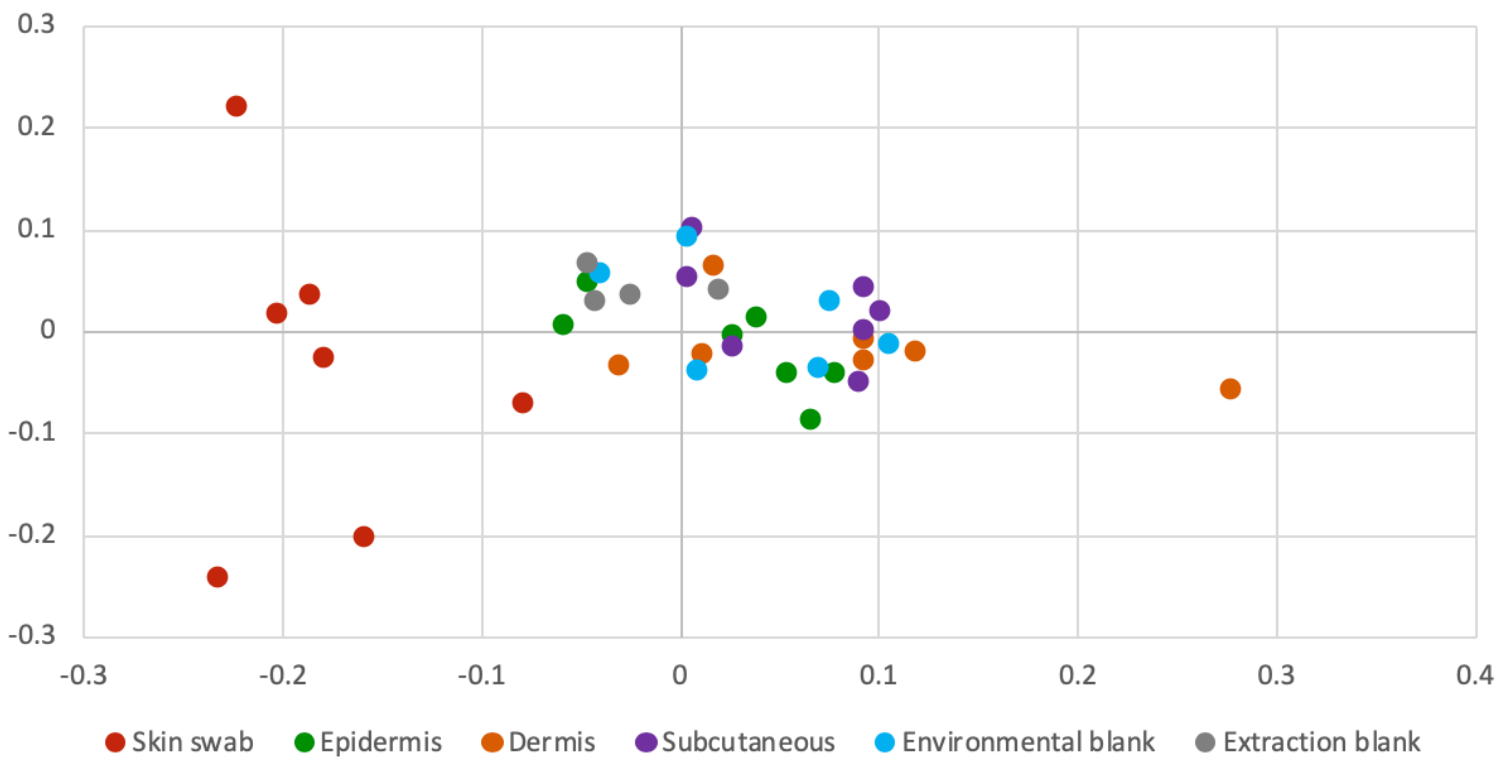

# PCoA Unweighted UniFrac

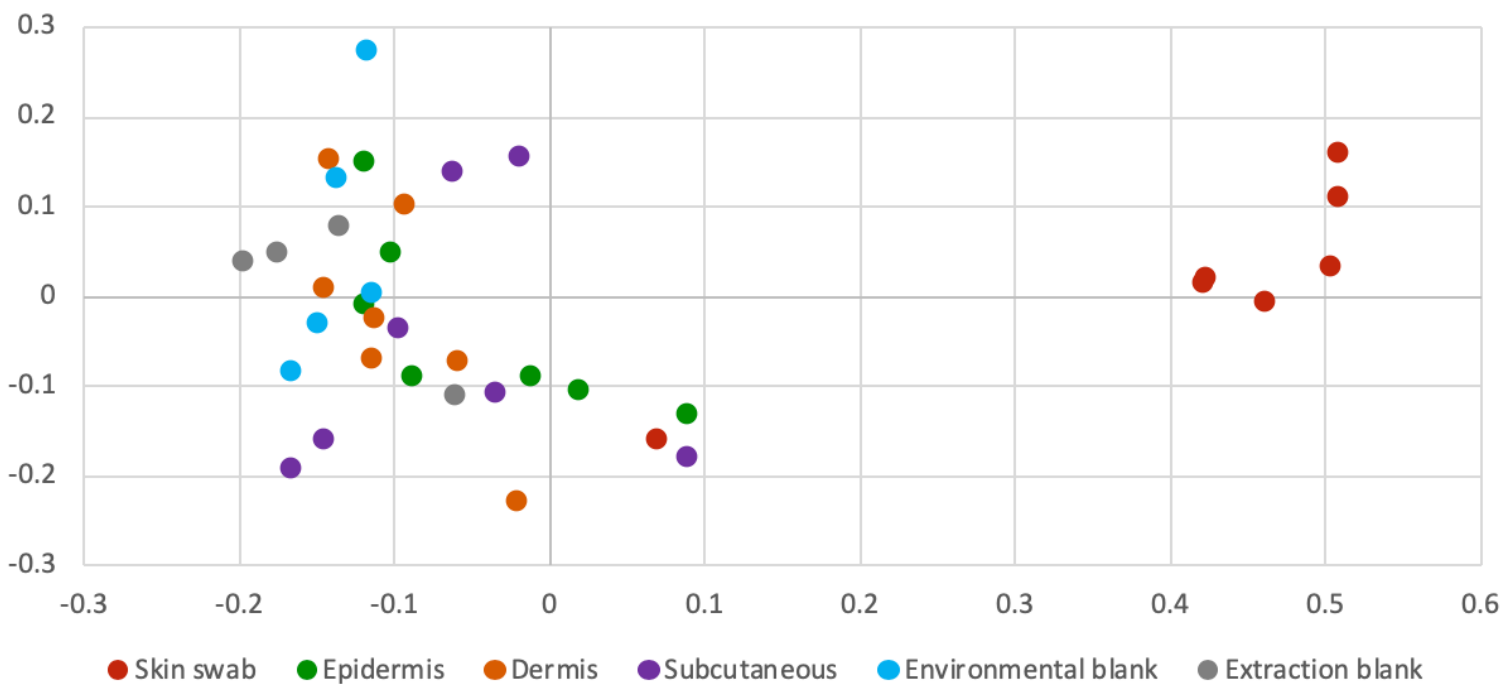

Supplement: Supplementary file 3 — Additional file 3. Unweighted and Weighted UniFrac PCoA plots. [file 42523_2020_50_MOESM3_ESM.pdf]

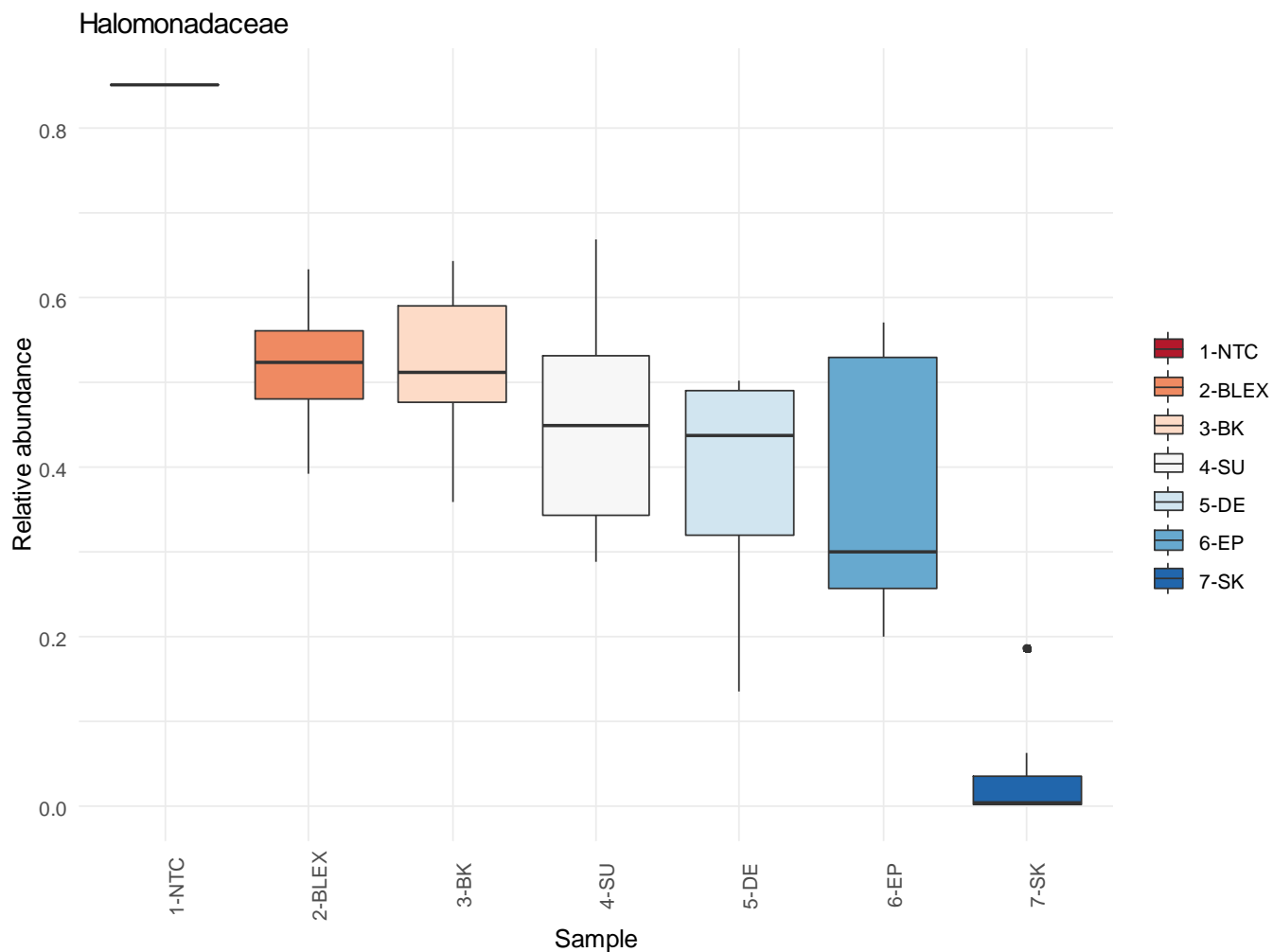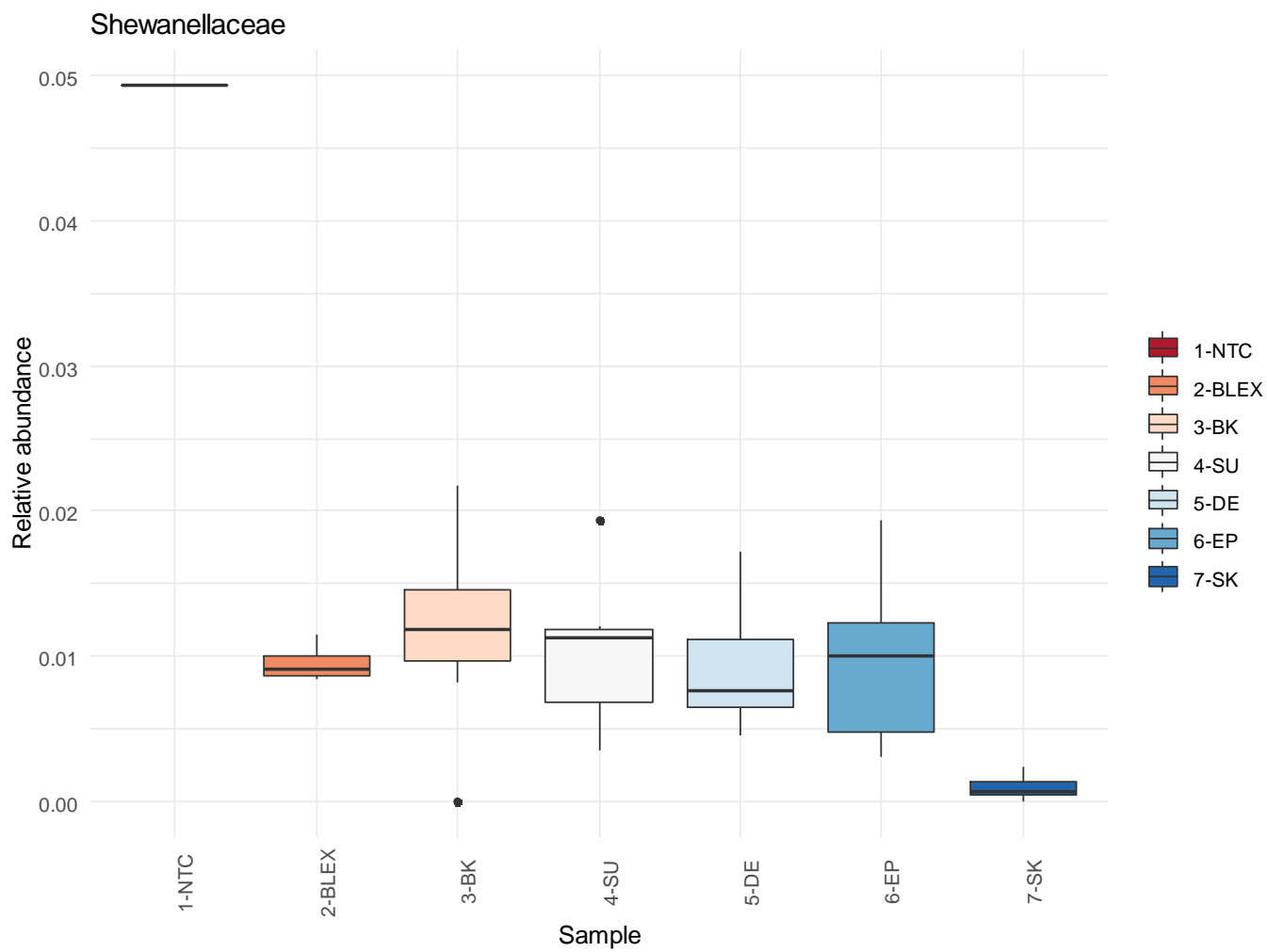

Supplement: Supplementary file 6 — Additional file 6 Boxplot representing the relative abundance of Halomonadaceae and Shewanellaceae on the different sample types. [file 42523_2020_50_MOESM6_ESM.pdf]
